# Cognition in children with arachnoid cysts – A five-year follow-up after microneurosurgical fenestration

**DOI:** 10.1007/s00701-024-06120-z

**Published:** 2024-05-22

**Authors:** Tomas Adolfsson, Erik Edström, Kristina Tedroff, Ulrika Sandvik

**Affiliations:** 1 Department of Clinical Neuroscience, Karolinska Institutet, 171 77 Stockholm, Sweden; 2 Department of Women’s and Children’s Health, Karolinska Institutet, 171 77 Stockholm, Sweden; 3 Department of Neurosurgery, Karolinska University Hospital, 17176 Stockholm, Sweden

**Keywords:** Arachnoid cysts, Children, Neurosurgery, Cognition, Long term follow-up

## Abstract

**Background and Purpose:**

In recent years there has been a re-evaluation regarding the clinical implications of temporal lobe arachnoid cysts (temporal arachnoid cysts) in children. These cysts have often been considered asymptomatic, or if symptomatic, only causing focal neurological symptoms or signs of increased intracranial pressure. However, several studies have more recently reported on cognitive symptoms improving after surgery. This study aimed to evaluate if reported cognitive improvement after surgery of temporal arachnoid cysts were stable after five years.

**Method:**

Ten consecutive children (m = 14.65; range 12.1–19.415 were assessed cognitively five years after micro-neurosurgical fenestration of a temporal arachnoid cyst. Results were compared to results from their pre- and post-surgical evaluations. Evaluations included the Wechsler-scales, Boston Naming Test (BNT), Rey Auditory Verbal Learning Test (RAVLT), verbal fluency test (FAS) and Rey Complex Figure Test (RCFT).

**Results:**

The analysis revealed significant postsurgical improvement compared to baseline on the Wechsler-scales measures of general intelligence (FSIQ), verbal abilities (VCI) and processing speed (PSI). Mean differences after surgery were 8.3 for FSIQ, (p = 0.026), 8.5 for VI (p =  < .01) and 9.9 for PSI (p = 0.03). There were no significant differences in mean test results when comparing postsurgical scores with scores five years after surgery, indicating long-term stability of improvements.

**Conclusion:**

The results indicate that affected cognitive functions in children with temporal arachnoid cysts improve after surgery and that the improvements remain stable five years later. The improvements and long term stability were also consistent with the experience of both parents and children. The findings provide a strong argument for neurosurgical fenestration of temporal arachnoid cysts in children.

## Introduction

Arachnoid cysts in children are benign, space-occupying, intracranial anomalies with a reported prevalence of up to 2.6% [3]. The origins of the cysts are not fully understood but they are often considered congenital, caused by a duplication of the arachnoid during fetal development. Another less commonly expressed hypothesis is that the cysts are secondary to trauma or infection [10]. The duplication of the arachnoid layers leads to the formation of a fluid-filled sac which may compress and dislocate adjacent structures including the brain parenchyma [45]. In children arachnoid cysts are often identified incidentally during the first decade of life, when the child is undergoing neuroimaging for other reasons [3]. The most common cyst location, seen in 47% of cases is the middle cranial fossa (temporal arachnoid cysts), where the cyst compresses or dislocates the temporal lobe and in many cases also ventral posterior parts of of the frontal lobe [3].

Though often considered asymptomatic, arachnoid cysts have been linked to symptoms like rapid head growth, headaches, seizures, increased intracranial pressure, increased head circumference, focal neurological symptoms and mental developmental delay [9]. During the last decades a number of studies have presented results indicating that arachnoid cysts may also cause cognitive symptoms.The majority of research regarding this have been conducted in older teenagers and adults. In one study, 55 patientswith arachnoid cysts (41 temporal arachnoid cysts) aged 16–70 years old, were assessed cognitively and results were compared to a healthy controlgroup. The patients with cysts performed worse on tests of visual retention, impulse control, visual scanning and sequencing [31]. In another study with 22 adult patients with supra-tentorial cysts (19 temporal arachnoid cysts) the patients performed worse than a control group in tests of verbal knowledge, mental flexibility, inhibitory capacity, problem solving, and planning skills [30]. This research have focused on arachnoid cysts in different locations.

More recent research have been conducted in children with cysts specifically located in the temporal lobe.Results indicate that children with temporal arachnoid cysts also present with cognitive symptoms. In a study by Cuny and co-workers 100 consecutive children with temporal arachnoid cysts were assessed cognitively [12]. The authors found that the children had lower results on the Wechsler-scales measure of general cognitive ability, verbal functions, processing speed and working memory. Impairments were also found in other tests of language, memory, executive functions and visual attention. In another study, 32 children with temporal arachnoid cysts were assessed with the Stanford Binet Intelligence Scale [23]. The results revealed significantly lower scores for general verbal knowledge, visuospatial functions and quantitative reasoning. In a previously published study from our team similar results were found in the presurgical assessment of 11 children with temporal arachnoid cysts. The children in our study had clearly below average preoperative scores on overall IQ, verbal comprehension and processing speed [33].

Alongside the growing knowledge of cognitive symptoms in children with temporal arachnoid cysts there are also studies reporting improvement in cognitive functions after decompression of the cysts. In a follow-up by Cuny [11] 34 of a initial 100 children that went through neurosurgery were re-assessed cognitively after surgery. The authors found statistically significant postsurgical improvement on the Wechsler-scales measures of overall IQ, verbal comprehension, processing speed. and perceptual reasoning. Improvement were also seen in language and memory functions. In another study [22] three boys with temporal arachnoid cysts were evaluated cognitively before and after neurosurgery. In the authors presurgical examination the results showed that the boys had lower scores in tests of general cognitive capacity, executive functions like attention and processing speed, when compared to the population norms. They also performed worse in tests of verbal memory. A postsurgical follow-up, conducted 3 to 6 months after surgery revealed improvements in general cognition, executive functions and verbal memory. The postsurgical assessment in our previous study [33] reported improvements in overall IQ, verbal comprehension and processing speed.

Temporal arachnoid cysts can be treated surgically, through microneurosurgical decompression. This reduces cyst size, intracystic pressure and mass effect. There is, however, a lack of consensus on whether or not the cysts should be treated surgically and what should be the indications for the procedure. The traditional standpoint is that temporal arachnoid cysts should be treated conservatively when asymptomatic or when symptoms are ambiguous. Surgery should only be proposed when patients present with signs of increased intracranial pressure, ascertained rupture with intracystic or subdural hemorrhage or risk thereof [5]. Cognitive symptoms have thus far not been considered as indicators for surgery [4, 40]. Research on both adults and children indicate that there may be cognitive symptoms in patients with temporal arachnoid cysts and that these improve after surgery and therefore the question is raised more frequently about whether these improvements also should be considered in a decision about surgery.

To the best of our knowledge no long term cognitive follow-up of surgically treated children with temporal arachnoid cysts has been done utilising the same set of comprehensive and psychometrically robust test-instruments in the presurgical, postsurgical and long-time follow-up. The aim of the present study was to evaluate if cognitive improvements after surgery of temporal arachnoid cysts in children are stable over a period of five years.

## Materials and methods

This was a longitudinal prospective study conducted at Astrid Lindgren Children’s Hospital, Karolinska University Hospital, a tertiary hospital in Stockholm, Sweden. In the initial study 15 consecutive children with temporal arachnoid cysts were evaluated cognitively 6 months before surgery. Of these 15 children 11 underwent microneurosurgical fenestration of the cyst and were re-evaluated cognitively 6 months after the surgery [33]. One child was lost during the five-year follow-up and all results for that child were excluded from all analyses in the present study. In the present study 10 of the surgically treated children were evaluated five years after surgery (6 boys and 4 girls) (Tables 1 and 2). We compared the five-year follow-up results to the results from presurgical and postsurgical evaluations in the same cohort. The mean test–retest interval between the postoperative evaluation and five-year follow-up was 55.1 months. The median age at study inclusion was 9.2 years (range 6.8–12.6) and at the five-year follow-up, the median age was 14.7 years (range 12.1–19.4).

**Table 1:** Patient characteristics

patient	gender	Cyst laterality	Galassi type	Age at surgery^a^	Cyst volume^b^		
					Preop	Postop	Five year
1	m	left	I	13	50	22	19
2	m	right	I	11	18	12	12
3	f	left	II	7	78	39	35
4	f	left	I	11	21	11	9
5	f	right	III	6	341	207	186
6	m	left	I	7	18	7	5
7	m	left	I	10	75	4	10
8	f	left	I	9	41	41	45
9	m	right	I	7	22	24	28
10	m	right	I	9	13	3	3

^a^age in years. ^b^ cyst volume in milliliters. Abbreviations: f: female. m: male

**Table 2:** Neurological symptoms and cognitive complaints at referral

	Reason for referral to neurosurgeon or neurologist^a^	Preoperative subjective cognitive or behavioral symptoms
1	Suspected Chiari I(negative)	Language difficulties and learning difficulties
2	Epilepsy	Slow cognitive tempo
3	Vomiting	Difficulties concentrating, affected WM
4	Scull trauma	Difficulties concentrating, language disorder
5	Scull deformity	Behavioral problems, outbursts
6	Visuoperceptual symptoms	Visuoperceptual difficulties, OCD
7	Headache, dizziness	ADHD
8	Pubertas preacox	Affected WM and concentration
9	Headache	Visuoperceptual symptoms
10	Seizure	No initial complaints

^a^All cysts found incidentally under initial neuroimaging. Abbreviations: WM: Working memory

OCD: Obsessive Compulsive Disorder. ADHD: Attention Deficit Hyperactivity Disorder

The children were evaluated by the same neuropsychologist (TA) on all three occasions with an identical set of standardized and age-adjusted cognitive tests.

## Evaluation methods

### Wechsler intelligence scale

Wechsler Intelligence scales [42] are used to assess cognitive functions. The test is individually administered and consists of fifteen subtests of which 10 is used to establish a full-scale IQ (FSIQ) and four indexes for comparison in this study; Verbal Comprehension index (VCI), Visuospatial Index (VSI), Working memory index (WMI) and Processing speed index (PSI). In earlier editions of the Wechsler Intelligence scales the VSI was called perceptual reasoning index (PRI). In the present study we use the term VSI. We used the fourth edition of the scales in the presurgical and postsurgical evaluations and WISC-V in the five-year follow-up. At the five-year follow-up, two participants were older than 16 years and thus were evaluated with the adult version of the Wechsler scales, WAIS-IV. We used the most current and age appropriate versions of the scales to ensure that the most recent norms were used. The scores of the three versions of the Wechsler scales can be considered equivalent. According to the tests technical manuals, the three versions can be considered measuring the same constructs with a high level of correlation between versions [13, 24, 42].

### FAS

FAS is a test of phonological verbal fluency. The test subject is required to produce as many words as possible in 60 s each for the letters F, A, and S [38]

### Boston Naming Test (BNT)

Boston Naming Test is a test of forced naming ability. The test subject is required to name nouns from 60 black-and-white pictures [38].

### Rey Complex Figure Test (RCFT)

Rey Complex Figure Test is a test of visuospatial memory, where the test subject is required to draw a copy of a complex figure and recall the figure from memory after a 3 min and a 30 min delay [38]

### Rey Auditory Verbal Learning Test (RAVLT)

The Rey Auditory Verbal Learning Test (RAVLT) assesses verbal memory. The test subject is required to remember 15 words read 5 times by the examiner, with recall after each repeated reading and after a 15-min delay [38]

## Statistical analysis

A one-way repeated measures ANOVA with Bonferroni adjustment for multiple comparisons was used to compare the pre-surgical, post-surgical and five-year follow-up-results of the assessments with the Wechsler scales. The Friedman test was used to compare results for FAS, BNT, RAVLT and RCFT due to non-normally distributed data. The Wilcoxon rank-sum was used in analysis of between-group differences in the Friedman test. Data were analyzed with SPSS, version 28. Statistical significance level was preset to p < 0.05.

## Results

### Wechsler intelligence scale

There were significant differences in mean test scores when comparing results from the presurgical, postsurgical and five-year follow-up evaluations. When comparing postsurgical follow-up with baseline (presurgical scores) there were significant differences in the results for FSIQ (F (2, 10) = 9.852, p = 0.001), VCI (F (1.38, 10) = 6.37, p = 0.02) and PSI (F(2,10) = 6.47 p = 0.01). These indexes all improved from a low average presurgical level to an average postsurgical level. Mean improvement in FSIQ was 8.3 points (95% CI 1.00–15.61, p = 0.026) and on the VCI 8.5 points (95% CI 3.19–13.81, p =  < 0.01). The largest improvement was detected in processing speed, with a mean difference of 9.9 points (95% CI 1.0–18.8, p = 0.03) between presurgical and postsurgical scores (Table 3).

**Table 3:** Comparison of presurgical and postsurgical results on the Wechsler-scales

	Presurgical	Postsurgical	Difference	
	M (SD)	M (SD)	M (95% CI)	*p*
FSIQ	84.40 (9.06)	92.70 (12.46)	8,3 (1.00–15.61)	0.03*
VCI	85.90 (14.45)	94.40 (13.71)	8,5 (3.19–13.81)	< 0.01*
VSI	98.40 (11.80)	104.20 (10.05)	5,8 (-4.24–15.84)	0.73
WMI	82.50 (10.17)	83.60 (16.22)	1,1 (-9.43–11.63)	1.00
PSI	81.90 (10.90)	91.80 (13.18)	9,9 (1.00–18.80)	0.02*

* Indicates significance. **Abbreviations**: SD: Standard Deviation. 95% CI: 95% Confidence Interval. FSIQ: Full Scale Intelligence Quotient. VCI: Verbal Comprehension Index. VSI: Visuospatial Index. WMI: Working Memory index. PSI: Processing Speed Index

When comparing the scores from the five-year follow-up with the scores from the postsurgical evaluation, no significant differences were found (FSIQ, (p = 1.00), VCI (p = 0.88), PSI (p = 1.0)), indicating stability of postsurgical improvements five years after surgery of TLACs in children. (Table 4).

**Table 4:** Comparison of post-surgical and five-year follow-up scores on the Wechsler-scales

	Postsurgical	Five year follow-up	Difference	
	M (SD)	M (SD)	M (95% CI)	*p*
FSIQ	92.70 (12.46)	94.00 (10.21)	1,3 (-5.04–7.64)	1.00
VCI	94.40 (13.71)	99.00(13.45)	4.6 (-7.50–16.70)	0.88
VSI	104.20 (10.05)	95.50 (9.72)	-8.7 (-18.29–0.90)	0.08
WMI	83.60 (16.22)	89.80 (8.32)	6.2 (-5.18–17.58)	0.43
PSI	91.80 (13.18)	91.80 (13.60)	0.00 (-9.61–9.61)	1.00

**Abbreviations**: SD: Standard Deviation. 95% CI: 95% Confidence Interval. FSIQ: Full Scale Intelligence Quotient. VCI: Verbal Comprehension Index. VSI: Perceptual Reasoning Index. WMI: Working Memory Index. PSI: Processing Speed Index

**Fig. 1:**
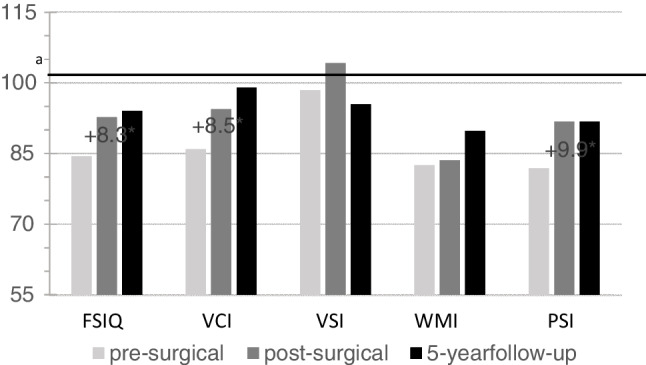
Preoperative, postoperative and five-year follow-up results on the Wechsler scales. FSIQ: Full Scale Intelligence Quotient. VCI: Verbal Comprehension Index. VSI: Perceptual Reasoning Index. WMI: Working Memory Index. PSI: Processing Speed Index. ^a^ population mean = 100, standard deviation = 15. Results ranging from 1 to 2 standard deviations below average are considered low average to clearly below average.^*^indicates significance

The results indicate that post-surgical improvements in cognitive test results remain stable after five years. The improvements in FSIQ, VCI and SI seen six months after surgery are still present after five years (Fig. 1). Results also indicate stability in the indexes that did not improve after surgery, indicating no deterioration in post-surgical results.

### FAS

No significant differences in results were detected between any of the three occasions for verbal fluency (χ^2^ (2) = 1.20, p = 0.55). The results on the test of verbal fluency were on an average level before and after surgery as well as at the five-year follow-up (Table 5).

**Table 5:** Comparison of scores from all three occasions on FAS, BNT, RCFT and RAVLT

		Presurgical				Postsurgical			Five-year follow-up			
	25th	50th	75th	25th	50th		75th	25th	50th	75th	χ^2^	*p*
FAS^a^	5.75	9.00	10.00	5.00	8.50		10.25	5,75	9.50	11.25	1,2	0.55
BNT^a^	1.92	5.38	8.02	1.95	4.32		7.88	1.27	3.28	5.92	2.65	0.27
RCFT^b^	26.43	36.50	39.90	24.75	31.95		47.33	39.05	45.15	49.70	2.60	0.27
RAVLT^a^	7.00	11.00	12.50	9.5	12.00		13.50	12.50	13.00	13.50	4.22	0.12

^a^ results presented as scaled score, ^b^ results presented as T-scores. Abbreviations: FAS: verbal fluency test. BNT: Boston Naming Test. RCFT: Rey Complex Figure Test. RAVLT: Rey Auditory Verbal Learning Test

### Boston Naming Test (BNT)

On the BNT the analysis detected no difference in results between the three occasions for picture naming (χ^2^(2) = 2.65, p = 0.27). The picture naming ability was on a presurgical level below average and remained on this level at both postsurgical and five year follow-up (Table 5).

### Rey Complex Figure Test (RCFT)

No difference in results were found between the three occasions for visual memory (χ^2^(2) = 2.60, p = 0.27). The visual memory ability was on a level below average presurgically and at the postsurgical follow-up. Five years after surgery a non significant improvement to the level “low average” were seen (Table 5).

### Verbal memory (RAVLT)

No significant differences were found between the results on the three occasions regarding verbal memory for a list of words (χ^2^(2) = 4.22, p = 0.12). The results for verbal memory was on an average level before surgery and remained on this level after surgery and at the five-year follow-up. (Table 5).

## Discussion

The present study is to the best of our knowledge the first five year follow-up of cognitive functions after surgery of temporal arachnoid cysts in children. The results showed that postoperative improvement on the Wechsler-scales measures of general intellectual ability (FSIQ), verbal comprehension (VCI) and perceptual speed (PSI) in children with temporal arachnoid cysts remained stable five years after surgery. Several previous studies, including one by our team, have presented postoperative cognitive improvement [11, 23, 26, 30, 33]. These preoperative and postoperative comparisons have however been done with a shorter test–retest interval of 3–14 months and long term follow-ups have been lacking. One study investigated long term stability using a parent questionnaire to perform a follow up after four years. The authors found that parents experienced that postoperative cognitive improvement were still present after four years [11]. In the present five year follow-up we used the same comprehensive set of psychometrically robust cognitive test instruments as in the preoperative and postoperative asssessments [33].

When comparing preoperative and postoperative test results, several studies have presented improvement in general cognitive abilities measured with cognitive test batteries like the Wechsler-Scales, Stanford-Binet or Ravens Progressive Matrices [2, 11, 43]. These studies also report postoperative improvement in specific tests of perceptual functions, working memory, picture naming, verbal fluency and verbal memory. In our study we did not find improvement in results on tests of specific functions, neither at the short-term follow-up, nor after five years. We observed these functions to be on an average to low average level before surgery, after surgery, and at the five year follow-up.

Improvement of cognitive functions in children with ACs after surgery have a great impact on their everyday functioning. General IQ (FSIQ) predicts school performance to a large extent [32], and for this reason it would seem fair to conclude that the increase in the children’s FSIQ in our study would have a substantial impact on their overall school achievements. General intellectual functioning is not a single and coherent entity, and it is often divided into several underlying functions. In the Wechsler-scales this is represented by a hierarchical model where the FSIQ is the major factor explaining a substantial part of the variation in results on underlying functions in the test (VCI, VSI, WMI and PSI) [8]. As FSIQ explains a substantial part of underlying functions, an increase in this is of great value. In the present study we found an improvement of the VCI in the Wechsler-scales. This is of significance as verbal functions are important for memory formation and learning and also for social interactions [16]. The improvement in results on the PSI has an impact on the overall cognitive performance as processing speed gives a person quicker access to other cognitive functions [17, 39].

For children, school achievments are closely linked to their perceived quality of life and affects mental and emotional well-being [1]. Factors such as emotional resilience, self-esteem, ability to manage stress, and access to mental health support services can impact their emotional well-being and overall quality of life. The function of the temporal lobe can have a significant impact on an individual's quality of life. Dysfunction or impairment in the region can lead to various challenges that may affect daily functioning and overall well-being. Even small improvement in temporal lobe function might have an impact on the individual. Since the postsurgical improvement seems to be stable over time, it is possible that early intervention will produce the greatest benefit for the child.

A possible explanation to similarities between studies regarding improvement in verbal abilities and executive functions like attentional control and processing speed may be found in theories of neuroanatomical distribution of these functions. Many important language functions are located in the temporal lobe, perisylvian structures and from early childhood also posterior lateral and ventral frontal lobe structures [41]. The ventral and posterior lateral parts of the frontal lobe are also involved in executive functions [21]. The localization of the temporal arachnoid cysts adjacent to these structures may be one explanation to why they interfere with language and executive functions. However, for both language and executive functions like processing speed, attention and goal directed behavior the theory of strict localization of functions is a simplification. The theory must be complemented by more complex explanations, involving larger functional networks consisting of cortical and subcortical areas connected by white matter tracts [37, 44]. Language and executive functions rely on the integrity of the frontoparietal and language networks [6, 7, 15, 27]. Affected language and executive functions in children with temporal arachnoid cysts may be explained by this network theory, as structural and functional connectivity are related and alterations or damage to the structural network may affect functional connectivity [19].

Cysts are thought to act as mass lesions within the closed intracranial compartment and affect the adjacent brain parenchyma through local compression and possibly also by raising the overall intracranial pressure [28]. One possible explanation to the reduced capacity in certain cognitive domains lies in a decrease in regional cerebral blood-flow (rCBF) in structures and networks adjacent to the cyst. There are studies using Single-photon emission computed tomography (SPECT) that supports this hypothesis [25, 36]. As a decrease in rCBF have a negative impact on cognitive functioning [29, 34], a restoration of the blood-flow might explain the postoperative improvement of functional symptoms found in patients with ACs. The hypothesis of consequenses of mass-effects was also investigated in a study of brain glucose utilization in a patient with a left-sided temporal arachnoid cyst [14]. Using PET (Positron Emission Tomography) the researchers detected hypo-methabolism in the parenchyma adjacent to the cyst and after shunt-placement the metabolic activity in the affected parenchyma was significantly improved. The improvement in metabolism correlated with improvement of language functions. Language functions are typically located in the affected left hemisphere and this might be one explanation to this improvement. More research is however needed regarding the neurobiological mechanisms behind cognitive symptoms in temporal arachnoid cysts.

The finding that improvement in cognitive functions persist after five years is important, as it indicates that surgery has a potential of being beneficial for children with temporal arachnoid cysts in a longer perspective. The fact that the functions that did not improve in this study remained stable after five years is equally important, as this indicates that surgery does not seem to negatively affect cognitive functions in a longer perspective. Although results are promising for a postoperative reduction of cognitive symptoms and long term stability of these improvements, the risks of performing neurosurgery must also be taken into consideration. Risks include CSF leakage, postoperative infection, hemmorhage or need for reoperation [4]. The rate of complications in surgery of intracranial cysts differ somewhat between surgical techniques and complication rates reported for microneurosurgical fenestration of the cyst are between 6 and 16% and for endoscopic fenestration 10% to 18%. For cystoperitonial schunting the rate of complications has been reported to be 5% [35]. Specifically for children the rate of complications after surgery of temporal arachnoid cysts have been reported as low and when reported the complications are often considered mild [18]. When cysts are symptomatic several variabels have traditionally indicated the need for surgery: focal neurological deficits, mass effect, location, affected CSF dynamics, headaches and seizures [20]. In line with the results of the present study, cognitive symptoms should be considered as an additional important factor influencing the decision about surgery. A thorough cognitive evaluation should be a part of the investigation before a decision about surgery is made.

## Limitations

The study was conducted on a small group of consecutive patients and the study had no controlgroup This of course limits the generalizability of the results and will have to be taken into consideration when interpreting the results. Our study group is heterogenous regarding age, cyst size and location which also might affect the external validity of the study.

## Conclusion

In our study improvement in general intellectual ability, verbal functions and processing speed seen after surgery of temporal arachnoid cysts in children sustain over a period of five years after surgery. The improvement may have a significant impact on school achievements and consequently also on possibilities in the future lives of the children. The persistence of the improvement over a long period suggests that surgery of temporal arachnoid cysts in children may be justified in part by these improvements. A reasonable conclusion would be that the potential risk of cognitive deficits in these children must be considered and cognitive testing should be a part of the investigation in this group of patients.

## Data Availability

Data from the present study can be made available upon reasonable request.
